# Proteolysis Targeting Chimeras for BTK Efficiently Inhibit B-Cell Receptor Signaling and Can Overcome Ibrutinib Resistance in CLL Cells

**DOI:** 10.3389/fonc.2021.646971

**Published:** 2021-05-13

**Authors:** Yamit Shorer Arbel, Ben-Zion Katz, Ronen Gabizon, Amit Shraga, Yotam Bronstein, Talia Kamdjou, Anat Globerson Levin, Chava Perry, Irit Avivi, Nir London, Yair Herishanu

**Affiliations:** ^1^ Sackler Faculty of Medicine, Tel Aviv University, Tel-Aviv, Israel; ^2^ Department of Hematology, Tel Aviv Sourasky Medical Center, Tel Aviv, Israel; ^3^ Department of Organic Chemistry, The Weizmann Institute of Science, Rehovot, Israel; ^4^ Immunology Research Laboratory, Tel Aviv Sourasky Medical Center, Tel Aviv, Israel

**Keywords:** PROTACs (proteolysis targeting chimeras), BTK - Bruton’s tyrosine kinase, CLL (Chronic Lymphocytic Leukemia), ibrutinib, BCR (B cell receptor) signaling

## Abstract

Proteolysis targeting chimeras (PROTACs) are small molecules that form ternary complexes between their target and E3 ligase, resulting in ubiquitination and proteasomal degradation of the target protein. Using our own designed Bruton’s tyrosine kinase (BTK) PROTAC compounds, we show herein efficient BTK degradation in chronic lymphocytic leukemia (CLL) cells. The reversible non-covalent compound (NC-1) was the most potent and therefore we focused on this PROTAC to investigate its subsequent effects on the BCR pathway. NC-1 decreased baseline BTK phosphorylation as well as activation of BTK and other signaling molecules downstream of the BCR pathway, following IgM engagement. These effects were also obtained in samples from CLL patients with clinical resistance to ibrutinib and mutations at C481. NC-1 treatment further decreased baseline CD69 surface levels, completely abrogated its upregulation following IgM activation, decreased CLL cells migration toward SDF-1 and overcame stromal anti-apoptotic protection. In conclusion, our results indicate that targeting BTK using the PROTAC strategy could be a potential novel therapeutic approach for CLL.

## Introduction

Chronic lymphocytic leukemia (CLL) is the most common leukemia in the western world (1). Although in recent years there has been considerable progress in the treatment options for CLL, a definitive cure is still only achievable with allogeneic stem cell transplantation (2). When considering the essential role of BCR signaling in CLL pathogenesis (3), this pathway has become a target for anti-CLL therapy. Small molecules directed against kinases of the BCR pathway show impressive clinical activity. One of these molecules is ibrutinib, an irreversible inhibitor of Bruton’s tyrosine kinase (BTK) which has a critical role in the amplification of the BCR signal. Nevertheless, due to side effects and resistance that emerges over-time, there is a need to develop novel approaches to target the BCR pathway (4–7).

Proteolysis-targeting chimeras (PROTACs), a novel strategy that utilizes the intracellular ubiquitin-proteasome system to induce targeted protein degradation, are receiving much attention in the field of targeted therapies. This technology is based on using hetero-bifunctional molecules that direct a ligand to bind with the target protein, linked with another ligand to recruit an E3 ubiquitin ligase. When the ternary complex (target-PROTAC-E3) is formed, the recruited E3 employs an E2 ubiquitin-conjugating enzyme to transfer ubiquitin to the surface of the targeted protein. This process leads to proteasomal degradation of the target protein (8–12).

PROTACs have a number of advantages over standard chemical inhibitors, including increased selectivity, inhibition of all target protein functions (13–16), longer lasting effects due to the need for a new synthesis of target (17) and induction of degradation by sub-stoichiometric concentrations of PROTAC (18). BTK is an established target for non-covalent PROTACs (19–24). Our previous results showed efficient BTK degradation with reversible covalent PROTACs, as well as their non-covalent and irreversible counterparts (25).

In this study we examined the effect of the PROTACs on BCR signaling, activation, migration and apoptosis in CLL cells. We show efficient inhibition of the BCR signaling pathway while using PROTACs in both wild-type (WT) and BTK mutated CLL cells. These results provide a basis for further preclinical study of BTK PROTACs as a novel strategy for CLL therapy.

## Materials and Methods

### Patients and Samples

Cells were obtained from peripheral blood samples donated by patients fulfilling the standard criteria for CLL after signing informed consent approved by the Tel-Aviv Sourasky Medical’s Institutional Review Board according to the Helsinki Accords (**Table 1**). Peripheral blood mononuclear cells (PBMC) were isolated by Ficoll density-gradient centrifugation. Viable frozen cells were kept in FCS containing 10% DMSO and stored in liquid nitrogen. Before use, frozen cells were thawed and cultured at 37°C, 5% CO2, in RPMI medium supplemented with 10% FCS, penicillin, streptomycin, and L-glutamine. The samples used contained more than 90% CLL cells.

**Table 1:** Patient characteristics.

Patient	Gender/ Age (y)	Binet stage	ALC(x10^9^/L)	IGHV mutational status	FISH/TP53
**CLL_01 **	M/67	A	102	M-IGHV	Del13q
**CLL_02**	M/72	B	203	UM-IGHV	Del11q
**CLL_03**	F/61	B	193	UM-IGHV	Del13q
**CLL_04**	F/64	C	194	M-IGHV	Del13q
**CLL_05**	M/76	C	117	M-IGHV	Del17p
**CLL_06 **	M/71	A	70	UM-IGHV	Del17p/TP53mut
**CLL_07**	M/56	B	76	UM-IGHV	Del17p/TP53mut
**CLL_08**	M/43	A	120	UM-IGHV	Del17p
**CLL_09**	M/57	B	66	UM-IGHV	Del11q
**CLL_10**	M/57	B	144	UM-IGHV	Del11q
**CLL_11**	M/70	B	87	UM-IGHV	Del11q
**CLL_12 **	F/71	B	121	UM-IGHV	Del13q
**CLL_13 **	M/52	A	146	UM-IGHV	Del11q
**CLL_14**	F/72	A	190	M-IGHV	Del13q
**CLL_15**	M/54	B	61	M-IGHV	Normal
**CLL_16**	M/76	A	59	M-IGHV	Del13q
**CLL_17 **	M/67	B	88	UM-IGHV	Normal
**CLL_18**	M/60	B	219	UM-IGHV	Del11q
**CLL_19** **C481Y-mutation**	M/64	C	33	UM-IGHV	Del11q/TP53mut
**CLL_20** **C481S-mutation**	M/60	C	71	UM-IGHV	Del17p/TP53mut
**CLL_21** **C481F-mutation**	F/54	C	40	UM-IGHV	Del17p/TP53mut
**CLL_22**	F/70	C	70	ND	ND

M, male; F, female; y, years; ALC, absolute lymphocyte count; M-IGHV, mutated IGHV; UM-IGHV-IGHV, unmutated IGHV; TP53mut, TP53 gene mutated; ND-no data.

### Antibodies and Reagents

ERK1/2, Phospho-ERK1/2 (Thr202/Tyr204), Akt (pan), phospho-Akt (S473),PLC γ2, pPLC γ2(Tyr1217),BTK, Phospho-BTK (Tyr223),Lyn(5G2), cleaved PARP (Asp214), CD79a, phospho-CD79a (Tyr182), Syk,phospho-Syk (Tyr525/526), SHIP1 and phospho-SHIP1 (Tyr1020) antibodies were from Cell Signaling Technology (Beverly, MA). Anti-SRC family (phospho Y418)-Phospho-Lyn (Y396) was obtained from Abcam (Cambridge,UK).Purified anti-human actin antibody was obtained from MP Biomedicals (Illkirch,France). Goat anti Rabbit IgG (H+L)-HRP conjugate and Goat anti Mouse IgG (H+L)-HRP conjugate and Goat F(ab’)2 anti-human IgM or IgG were from Jackson Immunoresearch Laboratories, (West Grove, PA). Dynabeads Human T-Activator CD3/CD28 were obtained from Thermo Scientific (Rockford, IL). All antibodies utilized in the study were used in concentrations according to the manufacturer’s instructions. Ficoll-Paque PLUS from GE healthcare (Uppsala, Sweden), dimethyl sulfoxide (DMSO) from Merck (Darmstadt, Germany), RPMI, fetal calf serum (FCS), Dulbecco’s phosphate buffered saline (PBS), L-glutamine and penicillin-streptomycin were from Biological Industries (Beit-Haemek, Israel). BTK PROTACs RC-2, IR-2 and NC-1 (**Supplementary Figure 1**) were designed as previously described (25).

### Targeting BTK in CLL Cells

CLL cells were incubated with PROTACs (RC-2, IR-2 and NC-1) at the indicated doses and time intervals at 37°C. The PROTACs were dissolved in DMSO, and controls were treated with DMSO accordingly.

### Western Blotting

CLL cells were lysed in RIPA lysis buffer (Cell Signaling Technology, Beverly, MA) containing phosphatase inhibitor cocktail 2 and protease inhibitor cocktail (Sigma-Aldrich, St. Louis, MO). Extract from cell lysates were separated on 4–15% Criterion™ TGX™ Precast Midi Protein Gel (Bio-Rad Laboratories) and transferred electrophoretically to nitrocellulose membrane (Bio-Rad Laboratories). The membranes were incubated with the designated antibodies and HRP conjugated secondary antibodies according to the manufacturer’s instructions. Bands were detected using MyECL Imager (Thermo Scientific, Rockford, IL).

### Flow Cytometry

For activation marker analysis, CLL and normal B-cells (5x10^6^ cells/mL, 500,000 cells per tube) were stained with APC Mouse Anti-human CD19 and PE Mouse Anti-human CD69 (BD Biosciences, CA, USA) and incubated for 30 minutes on ice. For normal T cell activation analysis, peripheral blood mononuclear cells were stained with APC Mouse anti-human CD3 and PE Mouse anti-human CD69 (BD Biosciences, CA, USA). Isotype controls were APC Mouse IgG1, κ and PE Mouse IgG1, κ (BD Biosciences, CA, USA). Cells were washed and then suspended in 0.5 mL PBS/1% FCS. For cell viability and apoptosis analysis, CLL cells (5x10^6^ cells/mL, 500,000 cells per tube) were stained with the Annexin V/propidium Iodide MEBCYTO^®^ Apoptosis Kit (MBL, Nagoya, Japan), according to the manufacturer’s instructions. In both assays samples were acquired by BD FACSCanto II and analyzed using BD FACSDiva software.

### Migration Assay

Peripheral blood CLL cells were cultured in 6-well dishes (5x10^6^ cells/mL in RPMI 10% FCS) and incubated with 100 nM NC-1 BTK PROTAC for 18 hours.DMSO treated cells served as controls. A total of 100 μL, containing 5 × 10^5^ cells, was added to the top chamber of a 6.5-mm diameter Transwell culture inserts (Costar, Cambridge, MA) with a pore size of 5 μm. Filters then were transferred to wells containing medium with 100 ng/mL SDF-1α (Merck). Wells containing medium without SDF-1α served as a negative control. The chambers were incubated for 2 hours at 37°C in 5% CO2. After this incubation, the cells in the lower chamber were suspended and divided into aliquots for counting. The experiments were performed in triplicates.

### Co-Culture Assay

CLL cells (10x10^6^ cells/mL) were co-cultured with HS-5 (ATCC^®^ CRL-11882™) in 20:1 ratio in 6-well dishes. The medium for co-culture was DMEM (ATCC^®^ 30-2002™) supplemented with 5% FCS and 1% penicillin-streptomycin. The cells were incubated with 100nM NC-1 PROTAC for 48 hours at 37°C. Proteins were extracted from cells treated with PROTAC and controls and analyzed by Western blotting.

### Statistical Analysis

In order to compare between the two paired groups, within each type of experiment, the Student’s t test was applied to compare the means of normal distributed dependent variables and the Wilcoxon Signed-rank test was applied in order to compare the distribution of non-parametric dependent variables. All statistical analyses were performed using GraphPad Prism 8.0 software (GraphPad Software, San Diego, CA, USA). A P-value of <0.05 was considered as statistically significant.

## Results

### Efficient PROTAC-Mediated BTK Degradation in CLL Cells

**Figure 1:**
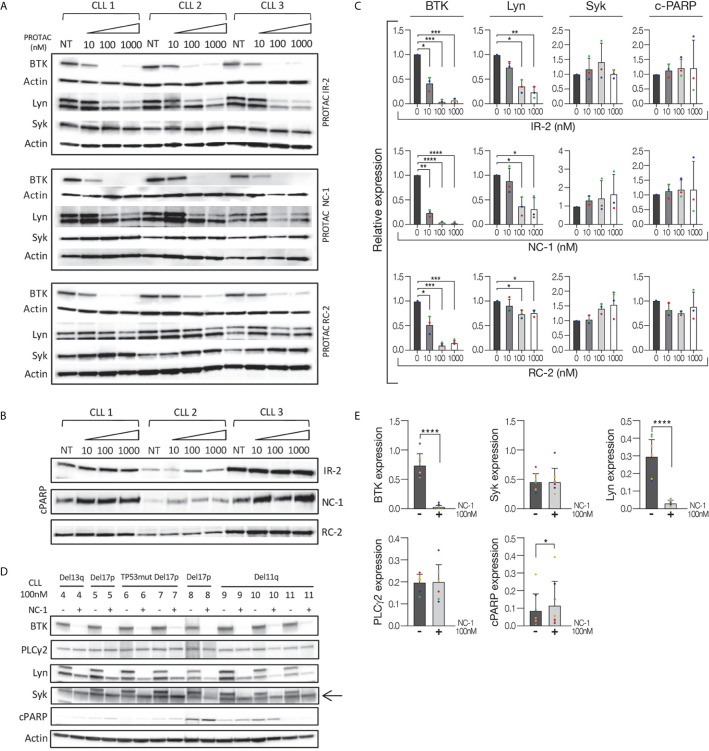
Efficient BTK degradation in CLL cells. Peripheral blood CLL cells of three patients were cultured in 6-well dishes (20x10^6^ cells/mL in RPMI 10% FCS) and incubated with BTK PROTACs in different concentrations (1000 nM, 100 nM and 10 nM) for 18 hours at 37° in a humidified 5% CO2 atmosphere. DMSO treated cells served as controls. Then, proteins were extracted and analyzed by Western blot. **(A)** A Western blot analysis showing total BTK, Lyn and Syk levels. Actin was used to verify equal loading. **(B)** Cleaved PARP levels in cells treated with different types and concentrations of PROTAC as a marker to measure apoptosis levels. **(C)** Quantification of BTK, Lyn, Syk and cleaved PARP levels in A and B by normalization to actin using myImageAnalysis™ Software (n=3). *p<0.05, **p<0.01, ***p<0.001, ****p<0.0001. **(D, E)** Peripheral blood CLL cells of 8 patients with various genetic abnormalities were cultured in 6-well dishes (20x10^6^ cells/mL in RPMI 10% FCS) and incubated with 100nM NC-1 for 18 hours at 37° in a humidified 5% CO2 atmosphere. DMSO treated cells served as controls. Then, proteins were extracted and analyzed by Western blot analysis. Levels of BTK, PLCγ2, Lyn, Syk and cleaved PARP (cPARP) are indicated (n=8). *p<0.05, ****p<0.0001.

We evaluated the ability of reversible covalent (RC-2), irreversible covalent (IR-2) and reversible non-covalent (NC-1) compounds (**Supplementary Figure 1**) to induce BTK degradation in CLL cell. The cells were treated for 18 hours based on our previous results using the three compounds in Ramos cells (25), as well as a time course experiment in CLL cells treated with 100 nM NC-1 PROTAC. Treatment with NC-1 decreased BTK in a time dependent manner and completely abolished BTK after 18 hours of incubation (**Supplementary Figure 2**). We also measured the expression levels of other proteins of the BCR pathway to test off-target effects. Proteins were extracted from cells treated with PROTACs and controls and analyzed by Western blotting. In our previous report (25), we showed that the three compounds decreased BTK levels, an effect that was more pronounced at the higher doses of 100-1000 nM. The NC-1 compound completely abolished BTK at ≥100 nM, and had a higher degradation potency compared to RC-2 and IR-2. In this work, total Lyn and Syk levels were also evaluated to confirm specificity of the PROTACs to BTK. Consistent with our previous report in the Ramos cell line, NC-1 and IR-2 also reduced Lyn levels, which is compatible with the known off-target effect of ibrutinib (26) (**Figures 1A, C**). Then, the effect of the most potent PROTAC, NC-1, at 100 nM was evaluated in 8 patients with different chromosomal abnormalities and/or *TP53* mutation. In all samples, the NC-1 compound led to BTK as well as Lyn degradation. Total Syk and PLCγ2 levels were not affected by this treatment (**Figures 1D, E**). In order to analyze the effect of the PROTACs on cell viability we tested cleaved PARP levels as a measure of apoptosis. Cleaved-PARP levels show a mild increase in apoptosis after 18 hours of PROTAC treatment (**Figures 1B–E**).

In order to validate the mechanism of PROTAC-mediated BTK degradation, CLL cells were treated with bortezomib, a proteasome inhibitor (27), for 1 hour before treatment with 100 nM NC-1. We analyzed BTK levels after additional 4 hours. PROTAC treatment was assessed for 4 hours due to the concomitant presence of bortezomib, which could cause cell death. Compatible with our previous results in Mino cells (25), bortezomib significantly inhibited degradation, suggesting proteasome-mediated degradation (**Supplementary Figure 3**).

### BTK PROTAC Leads to BCR Signaling Pathway Inhibition

**Figure 2:**
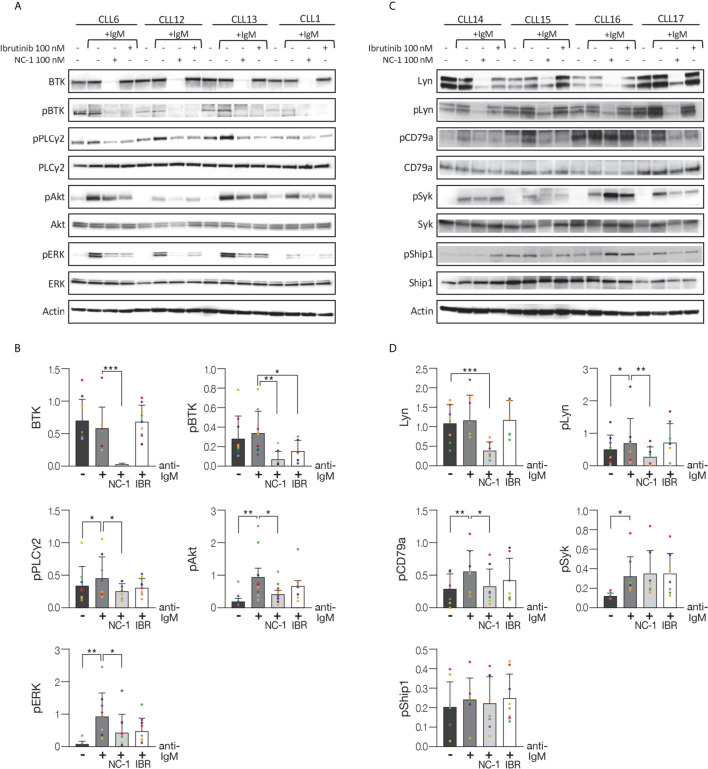
BTK PROTAC leads to BCR signaling pathway inhibition. Peripheral blood CLL cells were cultured in 6-well dishes (20x10^6^ cells/mL in RPMI 10% FCS) and incubated with 100 nM NC-1 for 18 hours or with ibrutinib (IBR) for 1 hour, at 37° in a humidified 5% CO2 atmosphere. DMSO treated cells served as controls. Following treatment, cells were incubated with goat F (ab’) 2 anti-human IgM (10 µg/mL) for 15 minutes or left unstimulated. Cells treated with ibrutinib were washed before activation. Then, proteins were extracted and analyzed by Western blot analysis. **(A)** A representative Western blot showing BTK (Tyr223), PLCγ2 (Tyr1217), Akt (S473) and ERK (T202/Y204) phosphorylation, as well as total amount of these proteins. Actin was used to verify equal loading. **(B)** Quantification of BTK, pBTK, pPLCγ2, pAkt and pERK levels in A by normalization to actin using myImageAnalysis™ Software (n=9). *p<0.05, **p<0.01, ***p<0.001. **(C)** A representative Western blot showing pLyn (Tyr396), pCD79a (Tyr182), pSyk (Tyr525/526) and pShip1 (Tyr1020) levels,as well as total amount of these proteins. Actin was used to verify equal loading. **(D)** Quantification of Lyn, pLyn, pCD79a, pSyk and pShip1 levels in C by normalization to actin using myImageAnalysis™ Software (n=8). *p<0.05, **p<0.01, ***p<0.001.

After demonstrating that the NC-1 PROTAC leads to effective BTK degradation, its effect on downstream elements in the BCR signaling pathway was tested. Peripheral blood CLL cells were treated with NC-1 or ibrutinib, and then activated with goat F(ab’)2 anti-human IgM. Cells that were not treated or stimulated served as controls. Western blot analysis revealed that pre-treatment with NC-1 resulted in abolishment of BTK phosphorylation, and partial inhibition of phosphorylation increase in Akt, ERK and PLCү2 following IgM cross-linking (**Figures 2A, B**). The inhibitory effect of ibrutinib on the BCR signaling was less prominent compared to that of NC-1 (**Figures 2A, B**). Since we have previously shown that Lyn is an off target of NC-1, we also analyzed the effect of this PROTAC on phosphorylation of proximal BCR elements. Treatment with 100nM NC-1 compound decreased Lyn levels, as well as phosphorylation of this protein and of CD79a after BCR activation (**Figures 2C, D**). The effect of NC-1 on Syk and Ship1 phosphorylation was heterogeneous and statistically non-significant, however in some samples (n=4) treatment with NC-1 clearly inhibited Syk phosphorylation after BCR activation (**Figures 2C, D**). The effect of ibrutinib on phosphorylation of the upstream signaling molecules of BCR pathway was not statistically significant (**Figures 2C, D**).

### Efficient BCR Signaling Pathway Inhibition in Ibrutinib Resistant CLL Cells

**Figure 3:**
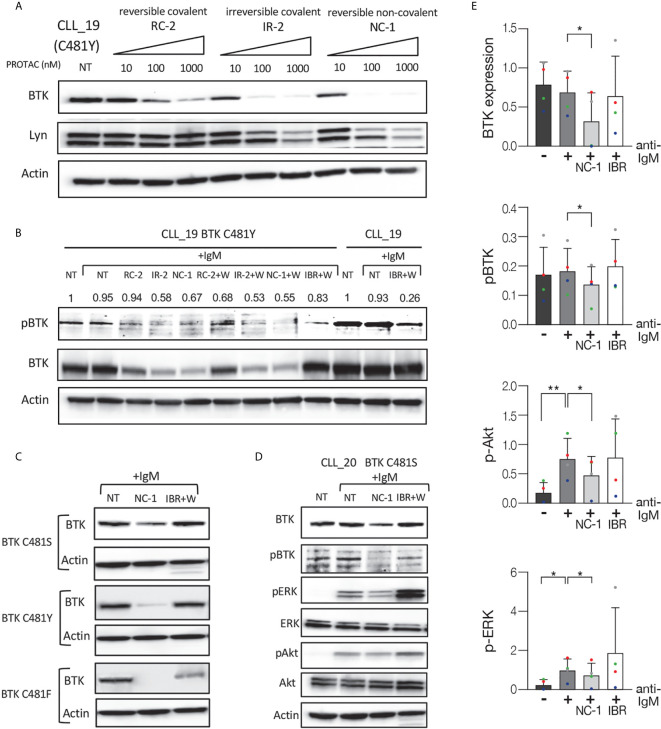
Efficient BCR signaling pathway inhibition in ibrutinib resistant CLL cells. Peripheral blood CLL cells of ibrutinib resistant patients were cultured in 6-well dishes (20x10^6^ cells/mL in RPMI 10% FCS) and incubated with BTK PROTACs for 18 hours or with ibrutinib (IBR) for 1 hour at 37° in a humidified 5% CO2 atmosphere. DMSO treated cells served as controls. Following treatment, cells were incubated with goat F(ab’) 2 anti-human IgM (10 µg/mL) for 15 minutes or left unstimulated. Cells treated with ibrutinib were washed before activation. Then, proteins were extracted and analyzed by Western blot analysis. **(A)** A representative Western blot showing BTK levels in C481Y CLL cells in response to various concentrations (1000 nM, 100 nM and 10 nM) of RC-2, IR-2 and NC-1 PROTACs. Total Lyn levels were evaluated to confirm specificity of the PROTACs to BTK. Actin was used to verify equal loading. **(B)** A Western blot analysis showing pBTK and BTK levels in response to treatment with 100 nM RC-2, IR-2, NC-1 or ibrutinib and activation with goat F(ab’)2 anti-human IgM in CLL cells before and after acquiring BTK C481Y mutation. PROTACs-treated cells were washed (W) or not washed prior to activation. **(C)** Total BTK levels in BTK mutated cells (C481S, C481Y and C481F) in response to 100nM NC-1 or ibrutinib. **(D)** A representative Western blot showing BTK (Tyr223), ERK (T202/Y204) and Akt (S473) phosphorylation, as well as total amount of these proteins, following treatment with 100 nM NC-1 or ibrutinib and activation with goat F(ab’)2 anti-human IgM in BTK C481S CLL cells. **(E)** Quantification of BTK, pBTK, pERK and pAkt levels in ibrutinib resistant cells (representative analysis, D) by normalization to actin using myImageAnalysis™ Software (n=4). *p<0.05, **p<0.01.

In the next experiments, we analyzed the effect of the PROTACs on the BCR signaling pathway in ibrutinib resistant cells. Ibrutinib binds BTK at the cysteine 481 residues and mutations at this position have been identified as the most frequent mutations in patients with CLL who develop clinical resistance. Peripheral blood CLL cells from patients with resistance to ibrutinib were treated with PROTACs or ibrutinib and then activated with goat F(ab’)2 anti-human IgM. Cells that were not treated or stimulated served as controls. Western blot analysis shows a substantial decrease in BTK levels in a dose-dependent manner in ibrutinib resistant cells (C481Y) treated with PROTACs (**Figure 3A**). In order to prove ibrutinib resistance, we analyzed its effect on phosphorylation levels of BTK in cells before and after acquiring the BTK C481Y mutation. It is important to emphasize that the mutations in BTK are heterozygous and in all experiments the cells from resistant patients were collected while patients continued ibrutinib treatment, and before further line of treatment. Because BTK mutations at C481 disrupt the covalent binding of ibrutinib to BTK, the cells treated with ibrutinib were washed before activation. As shown in **Figure 3B**, the relative decrease in pBTK levels after the cells were treated with ibrutinib was more prominent before the cells acquired the BTK C481Y mutation. Treatment with PROTACs led to a decrease in BTK and pBTK levels in cells resistant to ibrutinib (**Figure 3B**). We also show that there is no significant difference in the levels of BTK and pBTK between cells treated with PROTACs and washed before activation and those that were not washed (**Figure 3B**). The effect of the PROTAC NC-1 on BTK levels was also evaluated in cells with other BTK mutations, C481S and C481F. **Figure 3C** demonstrates BTK degradation in these ibrutinib resistant cells. Next, we analyzed the effect of NC-1 on downstream BCR signaling elements in cells from patients with ibrutinib clinical resistance. A representative Western blot analysis shows inhibition of the phosphorylation of BTK, Akt and ERK in cells treated with the PROTAC, an effect that was not observed when cells were treated with ibrutinib (**Figures 3D, E**). These results demonstrate that the PROTAC appears to be effective in degradation of BTK protein as well as inhibiting the downstream elements of the BCR signaling in BTK mutated cells.

### BTK PROTAC Blocks CLL Cell Activation in Response to BCR Engagement

**Figure 4:**
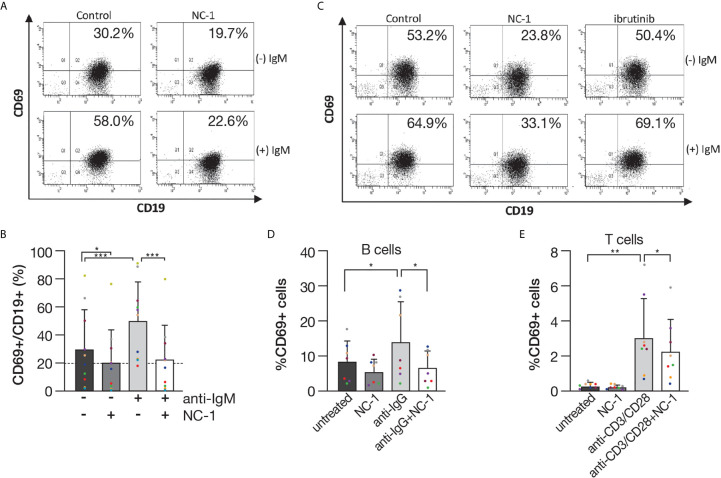
The effect of PROTAC on activation marker expression. Peripheral blood CLL cells were cultured in 6-well dishes (5x10^6^ cells/mL in RPMI 10% FCS) and incubated with 100 nM NC-1 BTK PROTAC for 18 hours or with 100nM ibrutinib for 1 hour at 37° in a humidified 5% CO2 atmosphere. DMSO treated cells served as controls. Following treatment, cells were incubated with goat F(ab’)2 anti-human IgM (10 µg/mL) for 3 hours or left unstimulated. After incubation, the cells were stained with APC Mouse Anti-Human CD19 and PE Mouse Anti-Human CD69 and incubated for 30 minutes on ice. Isotype controls were APC Mouse IgG1, κ and PE Mouse IgG1, κ. Samples were acquired by a FACSCanto II (BD) and analyzed using BD FACSDiva software. **(A)** Flow cytometric dot-plots of CD19-APC versus CD69-PE expression on samples of one representative CLL case. **(B)** Quantification of CD69+/CD19+ cells in samples obtained from CLL patients (n=10). *p<0.05, ***p<0.001. **(C)** Flow cytometric dot-plots of CD19-APC versus CD69-PE expression on samples of ibrutinib resistant CLL patient (C481S BTK). **(D, E)** Peripheral blood mononuclear cells (PBMC) isolated from healthy donors were incubated with 100 nM NC-1 for 18 hours. DMSO treated cells served as controls. Following treatment, cells were incubated with goat F(ab’)2 anti-human IgG (10 µg/mL) or with Dynabeads Human T-Activator CD3/CD28 for 3 hours or left unstimulated. After incubation, the cells were stained with APC Mouse Anti-Human CD19 or CD3, respectively, and PE Mouse Anti-Human CD69. Percentages of CD69+ in CD19+ (n=7) and in CD3+ (n=8) populations were calculated. *p<0.05, **p<0.01.

B-cell activation is generally accompanied by upregulation of cell surface expression of certain functional molecules. CLL cells present the phenotype of activated B cells based on the overexpression of the activation markers such as CD69, an early activation marker (28). CLL cells were treated with 100 nM NC-1, and then surface CD69 levels were determined by flow cytometry. As we expected, activation of the B cells with F(ab’) 2 anti-human IgM caused an increase in CD69 expression (**Figures 4A, B**). In cells treated with NC-1 there was a decrease in CD69 expression both at baseline and after activation (**Figures 4A, B**). NC-1 PROTAC treatment also decreased CD69 expression in BTK C481S mutated cells, whereas ibrutinib did not alter CD69 expression at baseline or after activation (**Figure 4C**). Our results show that PROTAC treatment blocks BCR-mediated activation in both WT and mutated BTK CLL cells. The effect of NC-1 compound on activation marker expression was also tested in normal B and T cells. The PROTAC decreased CD69 surface levels in both normal B and T cells after activation. However, there was no significant effect on this marker in resting cells treated with 100 nM NC-1 compound (**Figures 4D, E**).

### BTK PROTAC Induces Apoptosis, Inhibits Migration and Overcomes the Protective Effect of Stromal Cells on CLL Cells

**Figure 5:**
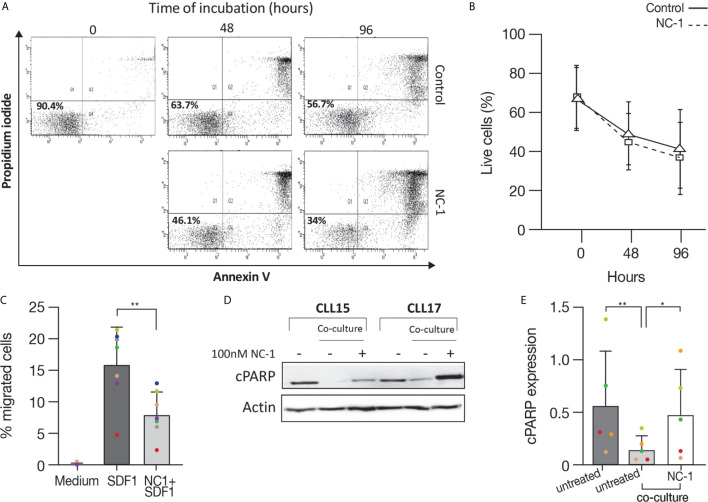
BTK PROTAC induces apoptosis, inhibits migration and overcomes anti-apoptotic protection of stromal cell on CLL cells. Peripheral blood CLL cells were cultured in 24-well dishes (5x10^6^ cells/mL in RPMI 10% FCS) and incubated with 100 nM NC-1 PROTAC at 37° in 5% humidified CO2 atmosphere. DMSO treated cells served as controls. Cells viability was evaluated by flow cytometry immediately after thawing, after 48 and 96 hours of incubation, using an Annexin V/PI apoptosis detection kit. **(A)** A representative flow cytometry analyses of annexin V-propidium iodide double staining CLL-treated cells and controls at 2 and 3 time points respectively. The left lower quadrant represents remaining live cells (percentages are indicated). The right lower quadrant represents the population of early apoptotic cells. The right upper quadrant represents the accumulation of late apoptotic cells. **(B)** Viability percentages of cells treated with PROTAC and controls (n=11) measured by flow cytometry after thawing, after 48 and 96 hours of incubation. **(C)** Peripheral blood CLL cells were cultured in 6-well dishes (5x10^6^ cells/mL in RPMI 10% FCS and incubated with 100 nM NC-1 BTK PROTAC for 18 hours. DMSO treated cells served as controls. A total of 100 μL, containing 5 × 10^5^ cells, was added to the top chamber of a 6.5-mm diameter Transwell culture inserts with a pore size of 5 μm. Filters then were transferred to wells containing medium with 100 ng/mL SDF-1α.Wells containing medium without SDF-1α served as a negative control. The chambers were incubated for 2 hours at 37°C in 5% CO2. After this incubation, the cells in the lower chamber were suspended and divided into aliquots for counting. The experiments were performed in triplicates (n=7). The averages + S.D. are shown. **p<0.01. **(D, E)** Peripheral blood CLL cells (10x10^6^ cells/mL) were co-cultured with HS-5 bone marrow stromal cells in 6-well dishes and treated with 100 nM NC-1. DMSO treated cells served as control. After 48 hours of incubation, proteins were extracted and analyzed by Western blot. A representative Western blot analysis showing cleaved PARP levels as a marker to measure apoptosis. Actin was used to verify equal loading (n=5). *p<0.05, **p<0.01.

After demonstrating the efficacy of PROTAC in suppressing elements in the BCR pathway and decreasing activation marker expression, we analyzed its effect on apoptosis. For this purpose, CLL cells were treated with 100 nM NC-1 PROTAC or left untreated. The concentrations of PROTAC used in the experiments are equivalent to the plasma therapeutic levels of ibrutinib in patients with CLL (29). Cells viability was determined by flow cytometry immediately after thawing, after 48 and 96 hours of incubation, using an Annexin V/PI apoptosis detection kit. As we expected, there was a decrease in cell viability over time. This effect was approximately 10% higher in cells that were treated with NC-1 BTK PROTAC (**Figures 5A, B**). The differences in cell viability between the treated cells and the control were statistically significant after 48 hours of incubation (P<0.049). These results confirm that BTK inhibition in the therapeutic range induces only mild apoptosis in CLL.

BTK has a role in mediating signal transduction that modulates migration and adhesion of B-cells to the tissue microenvironment, promoting cell survival and proliferation (30, 31). Given that fact, we analyzed the effect of NC-1 PROTAC on CLL cells migration toward stromal cell-derived factor-1 (SDF-1). It is already known that CLL cells express high levels of the chemokine receptor CXCR4 and that stromal cells secrete high amounts of SDF-1, thereby they can attract CLL cells *via* this receptor. This process can govern the homing and survival of CLL cells *in vivo* (31, 32). CLL cells were incubated with 100 nM NC-1 for 18 hours or left untreated. A total of 100 μL, containing 5 × 10^5^ cells was added to the top chamber of transwell culture inserts. Filters then were transferred to wells containing medium with 100 ng/mL SDF-1α. Wells containing medium without SDF-1α served as a negative control. After 2 hours of incubation, the cells in the lower chamber were counted. The results are the average ± S.D. of seven individual patients. The chemotaxis of CLL cells to SDF-1α was significantly inhibited by preincubation of the input cells with 100 nM NC-1 PROTAC (**Figure 5C**).

To study the capability of BTK PROTAC to overcome the supporting effect of mesenchymal stromal cells on CLL cells, CLL cells were co-cultured with HS-5, human bone marrow-derived stromal cells, and treated with 100 nM NC-1 for 48 hours. Proteins were extracted from CLL cells treated with PROTAC and controls and analyzed by Western blotting. Cleaved PARP levels were lower in CLL cells co-cultured with stromal cells compared to those incubated in medium alone. The addition of NC-1 to the co-culture system abrogated the anti-apoptotic effect of the stromal cells (**Figures 5D, E**). Taken together, these results demonstrate that PROTAC can overcome supporting effects of the tissue microenvironment on CLL cells.

## Discussion

In this study we demonstrate that the BCR pathway in CLL cells can be efficiently inhibited with PROTACs directed towards BTK. We show that non-covalent (NC-1), irreversible covalent (IR-2) and also reversible covalent (RC-2) PROTACs are able to degrade BTK in CLL cells. The NC-1 compound has a higher degradation potency compared with the IR-2 and RC-2 PROTACs. The efficacy of NC-1 was also observed in patients with poor genomic abnormalities, including del17p and *TP53* mutation. The higher potency of non-covalent NC-1 may be attributed to its rapid binding and dissociation equilibrium (25), as well as potentially improved stability and permeability. Furthermore, the non-covalent binding of NC-1 may explain its more prominent reduction of Lyn levels. While ibrutinib is a 400-fold weaker inhibitor of Lyn than BTK (26), efficient degradation of kinases by PROTACs was shown also based on weak binding inhibitors (13). Nevertheless, the reduction in Lyn levels can in fact have a beneficial therapeutic effect in CLL. Lyn plays a crucial role in the onset and progression of CLL and its targeting by dasatinib has been shown to inhibit BCR signaling in CLL cells (33).

Because of the higher NC-1 potency, we focused on this PROTAC to investigate its further effects on BCR signaling, activation, apoptosis and migration. In accordance with BTK degradation by NC-1, phosphorylation of BTK was abolished and activation of its downstream elements (PLCγ2, Akt and ERK) was partially blocked in response to IgM engagement. Although treatment with ibrutinib resulted in a reduction in BTK phosphorylation and also inhibition of BCR downstream elements, the latter effect was less prominent, to some extent, than that of NC-1. Given that Lyn is an off target of NC-1 compound, we also analyzed the effect of this PROTAC on phosphorylation of proximal BCR elements. The treatment led to decrease in phosphorylation of CD79a and Lyn, which can contribute to further inhibition of BCR pathway.

Treatment of ibrutinib-resistant CLL cells with mutations at C481 using NC-1 led to a decrease in BTK and pBTK levels, as well as in phosphorylation of Akt and ERK. As expected in BTK mutated cells, ibrutinib did not inhibit the phosphorylation of these elements. Taken together, our results demonstrate that the PROTACs are effective in degradation of BTK as well as inhibiting the downstream elements of the BCR signaling, including inhibitory activity in ibrutinib resistant cells with mutations at C481. From a therapeutic point of view, although PRTOACs have impressive activity in the targeting of BTK in CLL cells, over time compensatory molecules and pathways may arise that will oppose their activity, so in the future a combination of drugs should be studied.

CLL cells often present a phenotype of activated B cells with overexpression of surface activation markers including CD69 (28). Therefore, we analyzed the effect of the BTK PROTAC NC-1 on the expression of the early activation marker CD69. Treatment with NC-1 decreased the baseline CD69 surface levels, and completely abrogated the upregulation of CD69 following BCR activation both in BTK WT and mutated CLL cells. These results reinforce our findings that PROATCs directed to BTK are capable of blocking both “tonic” and “antigen-triggered” BCR signals in CLL cells. The effect of the PROTAC on this activation marker expression was also tested in normal B and T cells. As we expected, NC-1 compound decreased CD69 surface levels in activated normal B cells as they express BTK as part of their BCR complex. The PROTAC also decreased CD69 expression in activated normal T cells. This result can be explained by the possibility that NC-1 degraded the known ibrutinib off-target ITK in the T cells (26). In contrast to CLL cells, in normal cells there was no significant effect on CD69 levels in resting cells treated with 100 nM NC-1 compound. Our results indicate that in activated cells the effect of PROTAC is more pronounced and therefore it is an effective treatment strategy for CLL patients in whom the B cells have both “tonic” and “antigen –triggered” signals.

We also tested the effect of the PROTAC on apoptosis of CLL cells. Cleaved PARP analysis revealed a mild apoptosis after 18 hours of PROTAC treatment. Flow cytometry analysis using annexin V/PI staining revealed a decrease in cell viability after 48 and 96 hours of incubation, an effect that was approximately 10% higher in cells that were pre-treated with 100 nM NC-1 PROTAC, which is compatible with the mild apoptotic effect of ibrutinib using equivalent therapeutic plasma concentrations in CLL (50-100 nM) (29). It has been reported that ibrutinib can induce more marked apoptosis of CLL cells, but this has been achieved at supra-therapeutic concentrations up to 100 times higher than the concentration we used (6). These findings further support the concept that a central in-vivo mechanism of action of ibrutinib is displacement of CLL cells from their supportive microenvironment, leading to CLL cell redistribution from tissues into the blood, followed by cell death as a result of “death by neglect” (34).

It is already been previously published that CLL cells express high levels of CXCR4 and the stromal cells secrete SDF-1, thereby they can attract the CLL cells and subsequently govern their homing and survival (31, 32). BTK has a role in trafficking of CLL cells, and its inhibition results in impaired CXCR4 expression, signaling and function in CLL, as was seen while using ibrutinib (35, 36). In this study, the effect of NC-1 PROTAC on CLL cells migration toward SDF-1 was tested, using transwell migration assay. The chemotaxis of CLL cells to SDF-1α was significantly inhibited by preincubation of the cells with 100 nM NC-1 PROTAC. These results demonstrate that the PROTAC is effective in inhibiting an additional aspect of CLL cells physiology, which may cause *in vivo* blockade of CLL cell migration to secondary lymphoid tissues and bone marrow.

Given the importance of the stromal niche in CLL cells survival *in vivo*, the impact of PROTAC treatment on apoptosis in the presence of stromal support was investigated. Our results further show that NC-1 can overcome the anti-apoptotic protection of co-cultured stromal cells on CLL cells.

BTK PROTACs have already been developed (19–24) and the first designed BTK-targeted PROTACs were reported by Sun et al. (20). These studies showed a reduction in the level of BTK protein, inhibition of ERK phosphorylation in Ramos cell line (24), and degradation of BTK in CLL patient samples including C481S (22). The novelty of our work that we show the more comprehensive effects of PROTACs to BTK on proximal and downstream BCR signaling, activation, migration and apoptosis in patients derived CLL cells including those with different BTK mutations at C481. In addition, the use of PROTAC designed by us, has made it possible to further investigate the effect of compounds with different binding properties to BTK on CLL cells.

Taken together, PROTACs represent a very promising and powerful approach for the development of targeted therapy drugs, as was recently underscored by the first PROTAC, ARV-110, to enter clinical trials (12). For BTK, PROTACs such as NC-1 have advantages over irreversible inhibitors since they are not sensitive to mutations in C481 (**Figure 3**) as well as other reversible BTK inhibitors due to their longer duration of action. Furthermore, this work demonstrates the potential of BTK PROTACs to inhibit the BCR pathway, and paves the way for future development of this novel therapeutic modality in CLL. However, more efforts will be required to obtain deeper insight into the efficacy and safety of PROTACs in clinical settings.

## Data Availability Statement

The original contributions presented in the study are included in the article/**Supplementary Material**. Further inquiries can be directed to the corresponding author.

## Author Contributions

YSA, YH, and B-ZK designed the study, analyzed data, and wrote the paper. YSA performed and analyzed Western blot, flow cytometry and cell migration experiments. YH conducted statistical analysis. NL, AS, and RG designed the PROTACs. RG synthesized the PROTACs. All authors contributed to the article and approved the submitted version.

## Funding

YH is supported by grants from the Israeli Science Foundation (1707/19), Israeli Cancer Association and Sackler Faculty of Medicine, Tel-Aviv University. NL is the incumbent of the Alan and Laraine Fischer Career Development Chair. NL would like to acknowledge funding from the Israel Science Foundation (grant no. 2462/19), The Rising Tide Foundation, The Israel Cancer Research Fund, the Israeli Ministry of Science Technology (grant no. 3-14763), and the Moross Integrated Cancer Center. NL is also supported by the Helen and Martin Kimmel Center for Molecular Design, Joel and Mady Dukler Fund for Cancer Research, the Estate of Emile Mimran and Virgin JustGiving, and the George Schwartzman Fund. RG was supported by the state of Israel, ministry of Aliyah, Center for Integration in Science.

## Conflict of Interest

YH received honoraria from AstraZeneca and Janssen for work unrelated to the present study.

The remaining authors declare that the research was conducted in the absence of any commercial or financial relationships that could be construed as a potential conflict of interest.
