# Supplementary material for: Proteolysis Targeting Chimeras for BTK Efficiently Inhibit B-Cell Receptor Signaling and Can Overcome Ibrutinib Resistance in CLL Cells
Source: Front Oncol. 2021 May 13;11:646971. doi: 10.3389/fonc.2021.646971 (PMC8159153; doi:10.3389/fonc.2021.646971)
Supplement: Supplementary file 1 [file DataSheet_1.pdf]

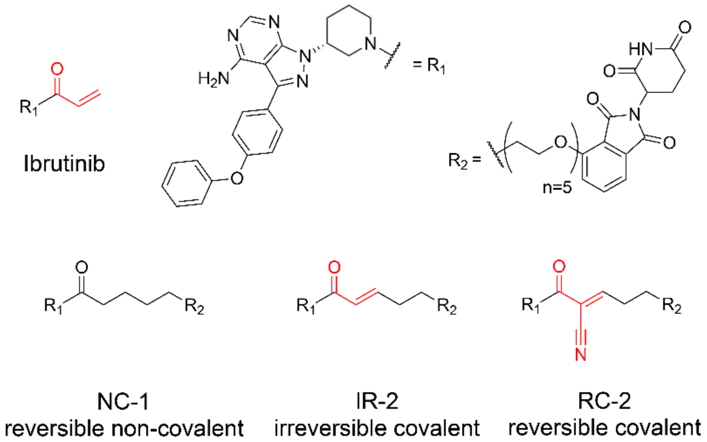

Supplementary Figure S1: structures of ibrutinib, reversible non-covalent, irreversible covalent and reversible covalent BTK PROTACs described in this study.

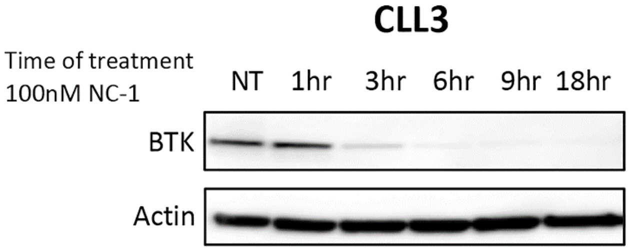

Supplementary Figure S2: BTK degradation time dependency. Peripheral blood CLL cells of 4 patients were cultured in 6-well dishes (20x10^6 cells/mL in RPMI 10% FCS) and incubated with 100 nM NC-1 BTK PROTAC for the indicated time intervals at 37° in a humidified 5% CO_2 atmosphere. DMSO treated cells served as controls. Then, proteins were extracted and analyzed by Western blot for BTK expression. A representative Western blot analysis showing BTK levels in CLL cells. Actin was used to verify equal loading.

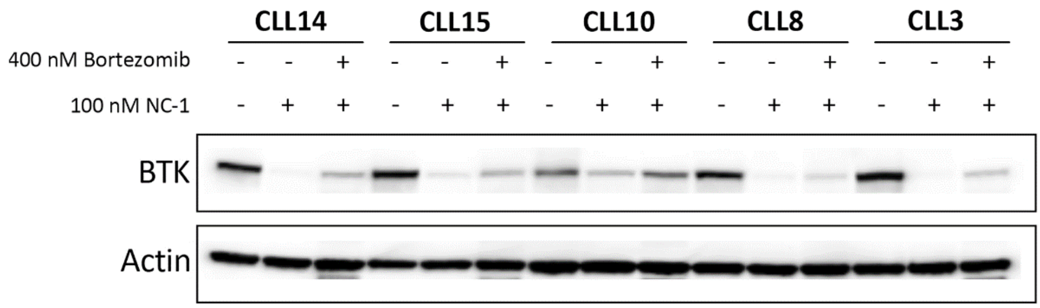

Supplementary Figure S3: PROTAC-mediated BTK degradation is hindered by proteasome inhibition. Peripheral blood CLL cells were cultured in 6-well dishes ($20x10^6$ cells/mL in RPMI 10% FCS) and treated for 1 hour with 400 nM bortezomib to inhibit proteasome-dependent degradation. Then, 100 nM NC-1 PROTAC was added for 4 hours. DMSO treated cells served as controls. Proteins were extracted and analyzed by Western blot for BTK levels. Actin was used to verify equal loading.
